# Characterization of lower-airway microbial communities in pulmonary tuberculosis by BALF metagenomic sequencing

**DOI:** 10.3389/fcimb.2026.1938581

**Published:** 2026-10-23

**Authors:** Hongxia Shao, Xue Li, Xuan Miao, Liang Tang, Yueying Gu, Zhonghua Qin, Junfeng Han, Jing Li, Qi Wang, Wanjie Yang, Huaiyong Chen, Junping Wu

**Affiliations:** 1 Department of Tuberculosis, Haihe Hospital, Tianjin University, Tianjin, China; 2 Tianjin Key Laboratory of Lung Regenerative Medicine, Haihe Hospital, Tianjin University, Tianjin, China; 3 Department of Radiology, Haihe Clinical School, Tianjin Medical University, Tianjin, China; 4 Department of Tuberculosis, Haihe Clinical School, Tianjin Medical University, Tianjin, China; 5 Center for Accurate Detection of Tuberculosis, Haihe Hospital, Tianjin University, Tianjin, China; 6 Key Research Laboratory for Infectious Disease Prevention for State Administration of Traditional Chinese Medicine, Tianjin Institute of Respiratory Diseases, Tianjin, China

**Keywords:** bronchoalveolar lavage fluid, metagenomic next-generation sequencing, mycobacterium tuberculosis complex, pulmonary microbiome, pulmonary tuberculosis

## Abstract

**Objective:**

Pulmonary tuberculosis (PTB) is an infectious disease caused by the *Mycobacterium tuberculosis* complex (MTBC). This study aimed to characterize the lower-airway microbiome in patients with PTB and compare it with that of patients without PTB (Non-PTB) undergoing bronchoscopy for pulmonary disease, in order to describe differences in microbial composition between the two groups and improve our understanding of pulmonary microbial ecology in tuberculosis.

**Methods:**

We performed metagenomic next-generation sequencing (mNGS) on bronchoalveolar lavage fluid (BALF) from 75 patients (38 PTB, 37 Non-PTB). Microbial diversity, composition, and differential taxa were analyzed using standard bioinformatics tools.

**Results:**

PTB samples showed higher Shannon and Simpson indices, whereas Richness did not differ significantly between groups. Beta diversity analyses revealed modest but statistically detectable differences in microbial community composition between PTB and Non-PTB samples. The PTB group showed higher relative abundances of MTBC and several oral-associated taxa (e.g., *Prevotella* and *Fusobacterium*), whereas Non-PTB samples showed higher relative abundances of taxa including *Pseudomonas aeruginosa* and *Aspergillus fumigatus*.

**Conclusion:**

This BALF metagenomic study characterized lower-airway microbial features in patients with PTB and identified differences in microbial community composition and structure between PTB and patients with heterogeneous Non-PTB pulmonary diseases.

## 1 Introduction

Pulmonary tuberculosis (PTB), caused by infection with *Mycobacterium tuberculosis* complex (MTBC), is a chronic infectious disease of the respiratory system and remains one of the leading communicable diseases posing a serious global public health threat (Berida and Lindsley, 2024). According to the World Health Organization (WHO) 2024 report, approximately 10.8 million new cases of PTB occurred worldwide in 2023, with more than 1.25 million deaths (World Health Organization, 2024). Although various pathogen detection methods have been established in clinical practice, early diagnosis of PTB remains challenging, particularly in cases with co-infection by other pathogens or where traditional diagnostic tools lack sufficient sensitivity (Dorman et al., 2018; Gunasekera et al., 2022; Zaporojan et al., 2024).

Recent studies have overturned the long-held belief that the lungs are sterile, revealing the presence of relatively stable microbial communities even in healthy individuals. The pulmonary microbiome plays a critical role in maintaining respiratory immune homeostasis, modulating host inflammatory responses, and contributing to disease progression (Hong et al., 2016; Yi et al., 2022; You et al., 2024; Wang B. et al., 2025). Alterations in lung microbial composition have been observed in PTB patients, suggesting that dysbiosis may be closely linked to immune dysregulation following MTBC infection (Hu et al., 2020b; Ding et al., 2021; Li et al., 2024). However, most current studies have focused either on the gut microbiome or on comparing healthy individuals with tuberculosis (TB) patients, and systematic comparisons between PTB and other non-tuberculous pulmonary disease remain scarce (Xiao et al., 2022; Zhao et al., 2023).

Metagenomic next-generation sequencing (mNGS), owing to its culture-independent, unbiased, and comprehensive nature, has been widely employed in the accurate diagnosis of infectious diseases and the profiling of pathogenic spectra. It is particularly advantageous for detecting multiple pathogens and analyzing microbiomes in complex respiratory infections (Gu et al., 2019; Miao et al., 2022). Comparative analysis of BALF microbial communities between PTB patients and patients without PTB (Non-PTB) undergoing bronchoscopy for various pulmonary diseases may help characterize differences in lower-airway microbial composition between PTB and other pulmonary diseases and improve our understanding of lower-airway microbial ecology in tuberculosis.

In this study, we employed mNGS to sequence bronchoalveolar lavage fluid (BALF) samples obtained from PTB and Non-PTB patients. We analyzed their microbial composition and diversity, compared differences in pulmonary microbiome structure between the two groups. Our findings aim to characterize the pulmonary microbial landscape associated with PTB and to provide a descriptive framework for future studies investigating disease-specific microbial patterns.

## 2 Methods

### 2.1 Study participants and sample collection

This was a retrospective observational study involving cases admitted to Tianjin Haihe Hospital between April 2022 and April 2024. Eligible participants were patients undergoing bronchoscopy and BALF collection due to suspected pulmonary infection. Inclusion criteria were: (1) hospitalization for “pneumonia” or “pulmonary shadow”; (2) no prior anti-infective treatment before bronchoscopy; (3) age ≥18 years; and (4) provision of written informed consent for participation and BALF sampling. Exclusion criteria included: (1) repeated antibiotic use within the past 6 months, defined as ≥3 courses of antibiotic treatment, each lasting ≥7 days; (2) immunosuppressive therapy within the past 3 months; (3) glucocorticoid therapy within the past 4 weeks; (4) concurrent severe renal, cardiac, oncologic, or other serious systemic diseases; and (5) co-infection with HIV or other communicable diseases. During bronchoscopy, lavage was directed to the radiographically involved lung segment identified on pre-procedural chest imaging. Sterile warm saline was instilled into the selected segment, and bronchoalveolar lavage fluid (BALF) was collected. Each BALF sample was split for routine microbial culture and metagenomic next-generation sequencing (mNGS) analysis. All participants provided written informed consent prior to enrollment. The study was approved by the Ethics Committee of Tianjin Haihe Hospital (Approval No. 2023HHKT-002).

Following bronchoscopy, all 75 patients were stratified into two groups based on comprehensive clinical evaluation and microbiological findings: the PTB group (n = 38) and the Non-PTB pulmonary disease group (n = 37). The diagnosis of PTB was established based on an integrated assessment of clinical presentation, radiographic findings, treatment response, and available tuberculosis-specific diagnostic evidence, including microbiological and molecular tests such as sputum smear, culture (sputum or BALF), and GeneXpert MTB/RIF assay, as well as supportive immunological evidence from T-SPOT.TB (**Supplementary Table S1**). PTB classification was determined independently of the BALF mNGS result. Patients fulfilling the above diagnostic criteria were classified as PTB even when MTBC reads were not detected by BALF mNGS. The Non-PTB group was heterogeneous and included bacterial pneumonia, viral pneumonia, Mycoplasma infection, and non-infectious pulmonary diseases, with all participants having no evidence supporting active MTBC infection (**Supplementary Table S2**).

### 2.2 Sample preparation and sequencing workflow

BALF samples were processed using a standardized mNGS workflow. Briefly, 800 μL of BALF was subjected to nucleic acid extraction using a magnetic bead-based extraction kit (Genskey Medical Technology Co., Ltd.), and nucleic acids were recovered in a final volume of 65 μL. DNA libraries were prepared using a UDB adaptor-based pathogen library preparation kit (Genskey Medical Technology Co., Ltd.) and sequenced on the MGISEQ-200 platform (BGI). Raw sequencing data were demultiplexed using bcl2fastq v2.20.0.422 and quality filtered using fastp v0.19.5 to remove adapter sequences, low-quality reads, and low-complexity sequences. Host-derived reads were removed by alignment to the human reference genome (GRCh38) using Bowtie2 v2.3.4.3. The remaining non-host reads were normalized to a reference library size of 20 million reads per sample for downstream microbial profiling and construction of the relative-abundance matrix. Per-sample sequencing read statistics, including raw reads, quality-filtered reads, host-derived reads, total microbial reads, and domain-level assignments to bacteria, viruses, fungi, and parasites, are provided in **Supplementary Table S3**, with corresponding cohort-level summary statistics provided in **Supplementary Table S4**.

A comprehensive in-house microbial reference database was constructed using representative genomes of bacteria, viruses, fungi, protozoa, and other eukaryotic microorganisms from the NCBI Nucleotide and Genome databases, comprising approximately 12,000 microbial genomes. Taxonomic classification was performed using genome alignment and the lowest common ancestor (LCA) algorithm based on NCBI taxonomy annotations. Species-level assignments were further filtered based on alignment specificity and genome coverage. Because BALF represents a low-biomass sample type, background contamination was assessed using batch-matched and historical negative controls, and taxa consistently detected in controls or below predefined abundance thresholds were removed. Microbial profiles were summarized at genus and species levels based on relative abundance. As absolute microbial loads were not measured, these data were interpreted as compositional differences rather than changes in microbial burden.

### 2.3 Bioinformatic analysis

Microbial taxa were ranked by their mean relative abundance across all samples, and the top 30 taxa were selected for visualization. The corresponding genus- and species-level microbial abundance profiles are provided in **Supplementary Tables S5**-**S10**. Alpha diversity indices, including Shannon, Simpson, and Richness, were calculated using the vegan package in R (**Supplementary Tables S11**, **S12**). Separately, read-based rarefaction curves were generated from raw taxon-assigned read counts to illustrate the relationship between sequencing depth and observed taxon richness (**Supplementary Figure S1**). Beta diversity was evaluated using Bray-Curtis dissimilarity through Principal Coordinates Analysis (PCoA), Non-metric Multidimensional Scaling (NMDS), and Analysis of Similarities (ANOSIM). The Bray-Curtis distance matrices and ordination coordinates are provided in **Supplementary Tables S13**-**S16**. Group-wise differences in taxonomic relative abundance were assessed using the Wilcoxon rank-sum test. Linear discriminant analysis effect size (LEfSe) was used as an exploratory analysis to identify taxa showing differential relative abundance between groups, using an LDA score >2.0 and a nominal P < 0.05 (**Supplementary Table S17**). Sensitivity analyses were additionally performed after exclusion of MTBC reads, and PTB samples were stratified according to the presence or absence of detectable MTBC reads for exploratory subgroup comparisons of alpha- and beta-diversity metrics.

### 2.4 Statistical analysis

Statistical analysis was conducted using SPSS software (version 26.0). A two-tailed p-value < 0.05 was considered statistically significant. Data normality was assessed using the Shapiro–Wilk test. Normally distributed continuous variables are presented as mean ± standard deviation (SD) and were compared using unpaired two-tailed t-tests or one-way ANOVA, as appropriate. Non-normally distributed data are shown as median with interquartile range [M (Q1, Q3)] and were analyzed using the Mann–Whitney U test or Kruskal–Wallis test. Categorical variables are expressed as percentages and compared using the chi-square test.

## 3 Results

### 3.1 Baseline demographics and clinical characteristics

A total of 75 patients were enrolled in this study, comprising 38 in the PTB group and 37 in the Non-PTB group (**Figure 1**). There were no statistically significant differences between the two groups in terms of age, sex, or most comorbidities (P > 0.05). However, the smoking rate was significantly higher in the Non-PTB group compared to the PTB group (48.6% vs. 23.7%, P = 0.024). No significant differences were observed in baseline clinical laboratory parameters between the PTB and Non-PTB groups, including liver function-related indicators such as alanine aminotransferase (ALT) and aspartate aminotransferase (AST). Detailed clinical characteristics are presented in **Table 1**.

**Figure 1:**
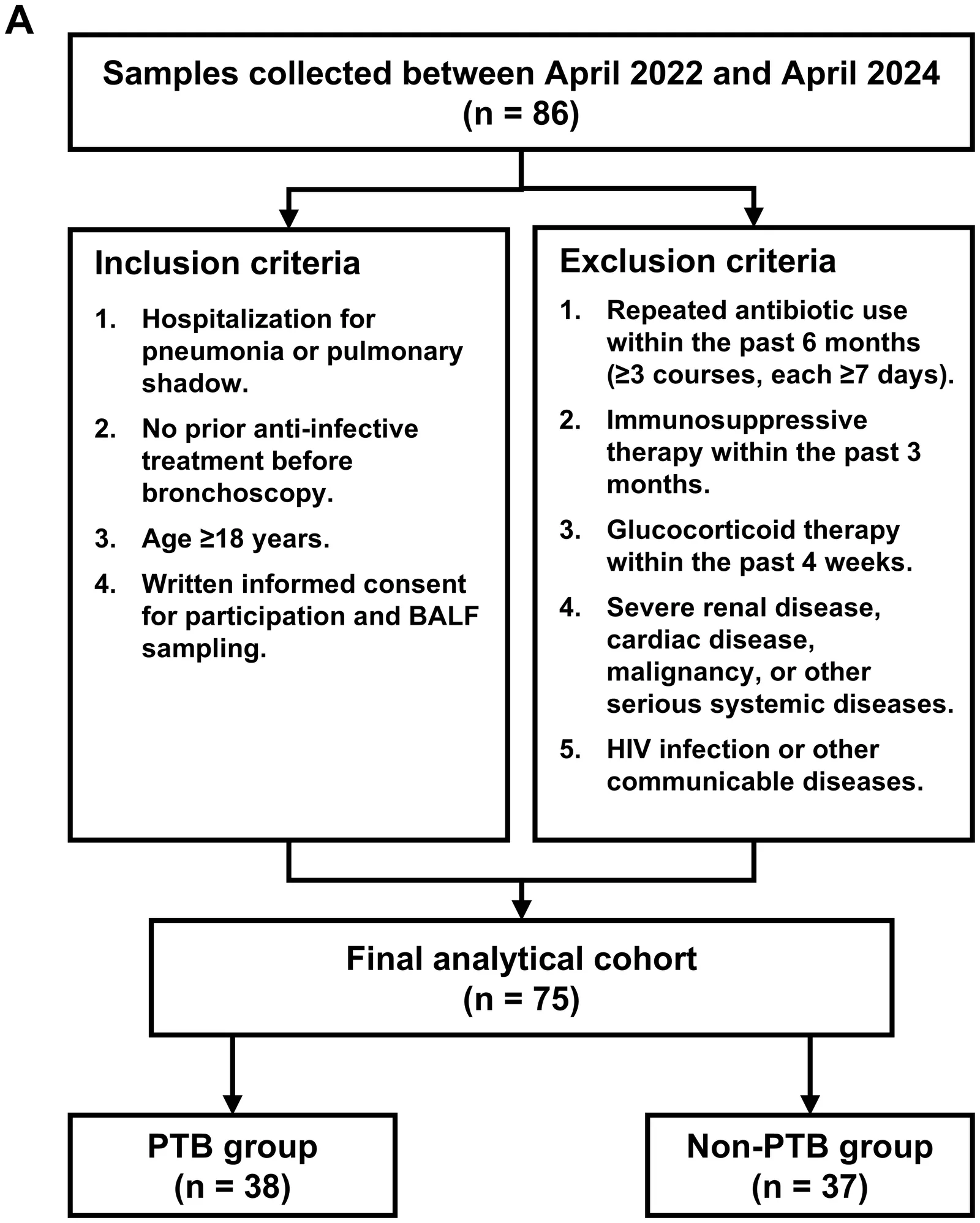
Participant flow diagram of study enrolment and final analytical cohort. Samples collected between April 2022 and April 2024 were screened according to the predefined inclusion and exclusion criteria. A total of 75 eligible participants were included in the final analytical cohort for microbiome analyses, comprising 38 patients in the PTB group and 37 participants in the Non-PTB group.

**Table 1:** Baseline demographic and clinical characteristics.

Characteristics	PTB (N = 38)	Non-PTB (N = 37)	*P*-value
Age, mean (SD),year	51.01 ± 18.49	59.05 ± 13.84	0.094
Gender, Female/Male (%)	18/20 (47.4/52.6)	14/23 (37.8/62.2)	0.404
Smoking, n (%)	9 (23.7)	18 (48.6)	0.024*
Drinking, n (%)	5 (13.2)	7 (18.9)	0.496
COPD, n (%)	1 (2.6)	3 (8.1)	0.291
Asthma, n (%)	1 (2.6)	1 (2.7)	0.985
Bronchiectasis, n (%)	6 (15.8)	2 (5.4)	0.145
Hypertension, n (%)	6 (15.8)	12 (32.4)	0.092
Diabetes, n (%)	7 (18.4)	10 (27)	0.373
Coronary heart disease, n (%)	4 (10.5)	3 (8.1)	0.719
WBC, median (IQR),*10^9^/L	6.42 (5.64, 7.75)	7.91 (5.46, 10.43)	0.097
NEU, median (IQR),*10^9^/L	4.23 (3.62, 5.41)	5.33 (3.66, 8.68)	0.059
LYM, mean (SD),*10^9^/L	1.38 ± 0.69	1.25 ± 0.75	0.437
HGB, median (IQR),g/L	127.5 (113.0, 140.0)	123.0 (112.0, 131.5)	0.314
TBIL, median (IQR),μmol/L	9.35 (8.35, 12.85)	11.9 (7.65, 14.25)	0.354
IBIL, median (IQR), μmol/L	8.00 (5.80, 10.30)	6.90 (4.00, 9.35)	0.221
ALT, median (IQR),U/L	19.10 (15.75, 27.13)	24.00 (16.95, 39.00)	0.151
AST, median (IQR),U/L	23.40 (16.63, 34.28)	25.00 (22.50, 35.50)	0.074
ALP, median (IQR),U/L	80.00 (61.00, 95.63)	80.00 (66.50, 119.25)	0.738
DD, median (IQR),mg/L	0.43 (0.27, 0.85)	0.50 (0.33, 1.24)	0.651

SD, standard deviation; IQR, interquartile range; COPD, chronic obstructive pulmonary disease; WBC, white blood cell; NEU, neutrophil; LYM, lymphocyte; HGB, hemoglobin; TBIL, total bilirubin; IBIL, indirect bilirubin; ALT, alanine aminotransferase; AST, aspartate aminotransferase; ALP, alkaline phosphatase; DD, D-Dimer. *p < 0.05.

### 3.2 Alpha diversity analysis

To assess within-sample microbial diversity, we compared Shannon, Simpson, and Richness indices between the two groups (**Table 2**; **Figures 2A–C**). At both genus and species levels, the PTB group (n = 38) exhibited higher Shannon and Simpson indices than the Non-PTB group, whereas the Richness index showed no significant difference between groups (P > 0.05). After exclusion of MTBC reads, the overall alpha-diversity pattern was maintained, with higher Shannon and Simpson indices in the PTB group and similar Richness between groups (**Supplementary Figure S2**). Alpha-diversity indices were also broadly comparable between PTB samples with and without detectable MTBC reads (**Supplementary Figure S3**).

**Table 2:** Alpha diversity analysis.

Species			
Group	Simpson	Shannon	Richness
PTB (n=38)	0.893 (0.653, 0.933)	2.804 (1.451, 3.194)	47.000 (23.750, 82.500)
Non-PTB (n=37)	0.639 (0.356, 0.848)	1.708 (0.993, 2.480)	51.000 (24.000, 94.000)
*P*-valu*e*	0.003**	0.012*	0.954
Genus			
Group	Simpson	Shannon	Richness
PTB (n=38)	0.807 (0.606, 0.863)	2.166 (1.448, 2.442)	37.000 (20.750, 62.500)
Non-PTB (n=37)	0.648 (0.398, 0.819)	1.658 (0.884, 2.138)	40.000 (17.000, 71.000)
*P*-value	0.012*	0.019*	0.870

**Figure 2:**
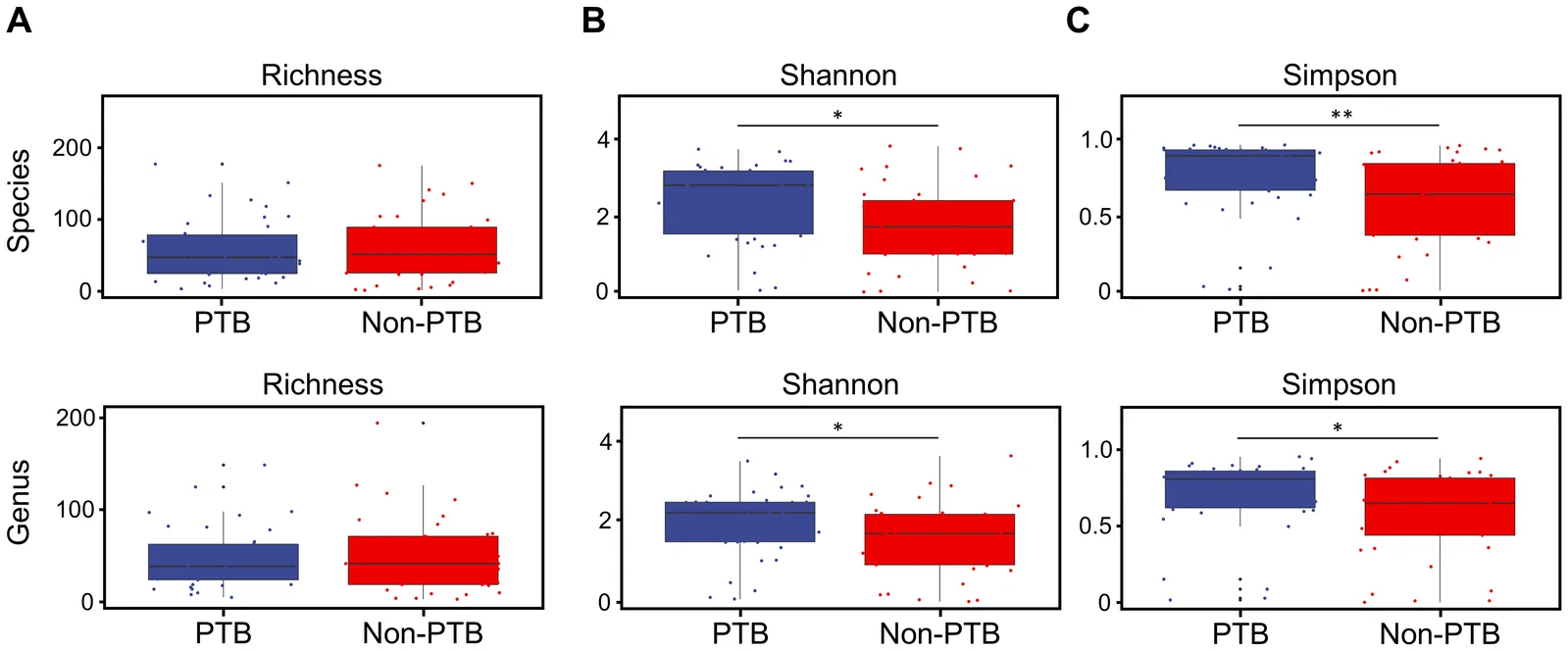
Alpha diversity analysis reveals intergroup differences between groups. The upper panels represent species-level data, while the lower panels show genus-level data. The x-axis indicates group classification, and the y-axis represents alpha diversity indices, including **(A)** Richness, **(B)** Shannon, and **(C)** Simpson indices. Boxes represent the interquartile range (IQR), with the horizontal line inside each box indicating the median. Statistical significance between groups was assessed using the Wilcoxon rank-sum test. *p < 0.05, **p < 0.01.

### 3.3 Beta diversity analysis

To evaluate differences in microbial community structure, ordination analyses were performed using Bray-Curtis distances (PCoA and NMDS, **Figures 3A, B**). Ordination analyses showed substantial overlap between PTB and Non-PTB samples. ANOSIM revealed R values of 0.094 at the species level and 0.090 at the genus level, with P = 0.001 for both comparisons, indicating modest but statistically detectable differences in microbial community composition between groups (**Figure 3C**). After exclusion of MTBC reads, substantial overlap between PTB and Non-PTB samples remained, while statistically detectable between-group differences were still observed by ANOSIM (**Supplementary Figure S4**). Furthermore, exploratory subgroup analyses showed no clear community-level difference between PTB samples with and without detectable MTBC reads (**Supplementary Figure S5**; **Supplementary Table S18**), suggesting that the observed community-level pattern was not solely attributable to the presence of MTBC reads.

**Figure 3:**
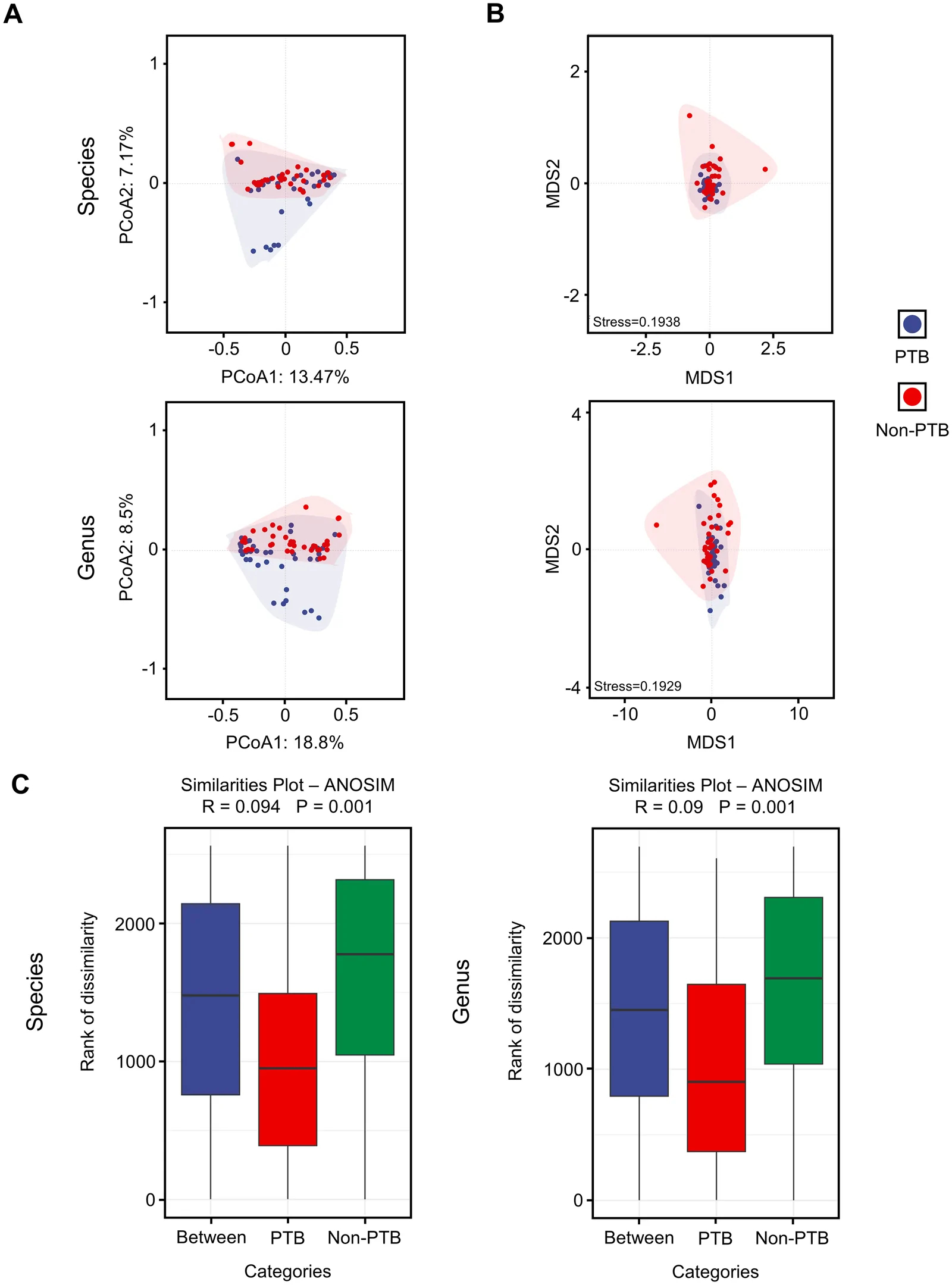
Beta diversity analysis of the pulmonary microbiota between groups. **(A)** Principal coordinates analysis (PCoA) and **(B)** non-metric multidimensional scaling (NMDS) based on Bray-Curtis distances were performed at both the species (top) and genus (bottom) levels. Each dot represents a sample, with blue and red indicating PTB and Non-PTB groups, respectively. Shaded areas denote 95% confidence ellipses. **(C)** ANOSIM showed modest but statistically detectable differences in microbial community composition between PTB and Non-PTB samples at both the species (R = 0.094, P = 0.001) and genus (R = 0.090, P = 0.001) levels.

### 3.4 Composition of pulmonary microbial communities

To characterize the microbial composition in BALF samples from both groups, we identified and visualized the top 30 genera and species based on relative abundance (**Figures 4A, B**). To further illustrate between-patient heterogeneity, sample-level relative-abundance profiles of the top 10 species are provided in **Supplementary Figure S6**. At the species level, MTBC accounted for 45.60% of the group-level relative abundance in the PTB group, followed by *Klebsiella pneumoniae* (8.83%), *Candida albicans* (4.64%), and *Prevotella melaninogenica* (3.49%). In contrast, the Non-PTB group had higher relative abundances of *Pseudomonas aeruginosa* (22.02%), *Rothia mucilaginosa* (12.69%), *Peptostreptococcus stomatis* (8.49%), *Parvimonas micra* (8.01%), and *Prevotella melaninogenica* (7.81%).

**Figure 4:**
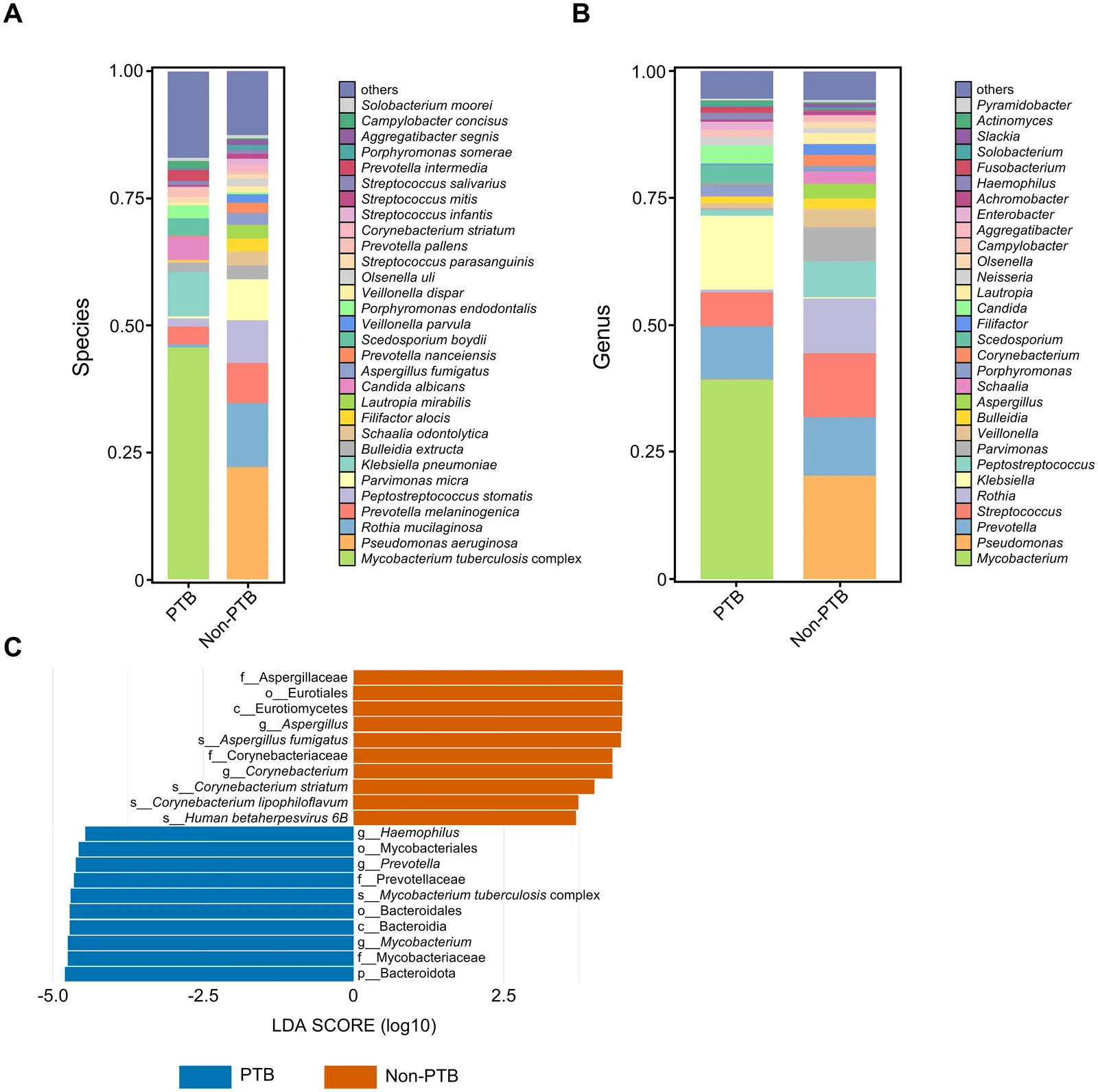
Taxonomic composition and differential microbial features between groups. Relative abundance of microbial taxa at the species **(A)** and genus **(B)** levels. Stacked bar plots show the proportion of dominant taxa in each group, with colors representing different taxa. **(C)** Linear discriminant analysis (LDA) showing the top 10 taxa with differential relative abundance ranked by LDA score between the PTB and Non-PTB groups. Bars indicate taxa identified in the exploratory LEfSe analysis, colored by group (blue for PTB and orange for Non-PTB). Complete LEfSe results are provided in **Supplementary Table S17**.

At the genus level, *Mycobacterium* showed the highest relative abundance in the PTB group (39.25%), followed by *Klebsiella* (14.54%), *Prevotella* (10.55%), and *Streptococcus* (6.66%). In the Non-PTB group, *Pseudomonas* was the most abundant genus (20.40%), followed by *Streptococcus* (12.68%), *Prevotella* (11.41%), and *Rothia* (10.70%). While *Prevotella* and *Streptococcus* were among the top taxa in both groups, notable differences were observed in the dominant genera between PTB and Non-PTB groups, indicating differences in the relative distribution of dominant microbial taxa between PTB and the heterogeneous non-PTB comparator group.

### 3.5 Differential abundance analysis between groups

Exploratory LEfSe analysis was performed to identify taxa showing differential relative abundance between groups. The top 10 taxa ranked by LDA score are shown in **Figure 4C**, the corresponding cladogram is provided in **Supplementary Figure S7**, and the complete LEfSe results are listed in **Supplementary Table S17**. Using an LDA score threshold of >2.0, species showing higher relative abundance in the PTB group included the *Mycobacterium tuberculosis* complex, *Prevotella melaninogenica*, *Haemophilus parainfluenzae*, *Fusobacterium periodonticum*, *Filifactor alocis*, and *Gemella haemolysans*. Species showing higher relative abundance in the Non-PTB group included *Aspergillus fumigatus*, *Corynebacterium striatum*, *Veillonella dispar*, *Acinetobacter baumannii*, *Ruminococcus gnavus*, *Prevotella oris*, and *Gemella morbillorum*. At the genus level, in addition to *Mycobacterium*, *Porphyromonas* and *Citrobacter* showed higher relative abundance in the PTB group.

## 4 Discussion

In this study, we employed mNGS to analyze the pathogen spectrum and microbial community structure of BALF samples from 38 PTB patients and 37 Non-PTB patients. We profiled the species composition and relative abundance of pathogens in both groups and conducted comparative analyses to characterize differences in pulmonary microbial composition between the PTB and Non-PTB groups. These analyses provide a descriptive comparison of the lower-airway microbial communities in the two groups.

Among samples meeting diagnostic criteria for active tuberculosis, 12 in the PTB group showed no detectable MTBC sequences. The Non-PTB group included patients with bacterial pneumonia, fungal pneumonia, viral pneumonia, nontuberculous mycobacterial pulmonary disease, lung cancer, and other pulmonary diseases. Demographic and clinical characteristics were generally comparable between the two groups, except for smoking prevalence, which was higher in the Non-PTB group than in the PTB group (48.6% vs. 23.7%, P = 0.024). This imbalance represents a potential source of confounding in the observed microbiome differences between groups.

Alpha-diversity analyses showed higher Shannon and Simpson indices in the PTB group, whereas Richness did not differ significantly between groups. Beta-diversity analyses based on Bray–Curtis distances showed substantial overlap between PTB and Non-PTB samples, while indicating modest but statistically detectable differences in microbial community composition at both the species and genus levels (Xiao et al., 2022; Liu et al., 2023). Sensitivity analyses excluding MTBC reads showed broadly consistent community-level patterns, and exploratory subgroup comparisons revealed no clear divergence between PTB samples with and without detectable MTBC reads. These findings suggest that the observed between-group differences were not solely attributable to the presence of MTBC reads.

At the genus level, *Prevotella* and *Streptococcus* were abundant in both groups. These genera are commonly associated with the oral microbiota and have also been reported in lower-respiratory tract samples, with microaspiration proposed as one route by which oral bacteria may reach the lower airways (Hu et al., 2020a; Kononen and Gursoy, 2022; Tett et al., 2021; Sun et al., 2023; Stankovic, 2025). The present study did not assess the route of entry or persistence of these taxa. Additional genera with relatively high abundance in the PTB group included *Mycobacterium* and *Klebsiella*. In contrast, the Non-PTB group showed higher relative abundances of *Pseudomonas* and *Rothia*, which have been associated with healthcare-associated infections and oral-associated microbial communities (Pathak et al., 2021; Reynolds and Kollef, 2021; Lin et al., 2024).

At the species level, the PTB group showed higher relative abundances of *Mycobacterium tuberculosis* complex (MTBC), *Klebsiella pneumoniae*, *Candida albicans*, and *Prevotella melaninogenica*, whereas *Pseudomonas aeruginosa*, *Rothia mucilaginosa*, *Peptostreptococcus stomatis*, and *Parvimonas micra* showed higher relative abundances in the Non-PTB group. Exploratory LEfSe analysis identified several taxa showing differential relative abundance between groups. MTBC, oral anaerobes (e.g., *Fusobacterium periodonticum* and *Filifactor alocis*), and conditional pathogens (e.g., *Prevotella melaninogenica* and *Haemophilus parainfluenzae*) showed higher relative abundance in the PTB group. These observations should be interpreted descriptively, as the present study did not directly evaluate the biological mechanisms underlying these compositional differences (Lin et al., 2024; Wang C. et al., 2025). In contrast, the Non-PTB group showed higher relative abundances of taxa such as *Aspergillus fumigatus* and *Acinetobacter baumannii*, reflecting differences in lower-airway microbial composition between the two groups (Keskilidou et al., 2025). Notably, *Klebsiella pneumoniae* and *Candida albicans* were relatively abundant in PTB but were not identified as significant differential taxa by LEfSe. Their detection may reflect airway colonization or co-infection in a subset of patients, and BALF mNGS relative abundance alone cannot distinguish colonization from active infection.

The Non-PTB comparator represented a clinically heterogeneous group that included bacterial, viral and non-infectious pulmonary diseases. Accordingly, some observed taxonomic differences, particularly the high relative abundance of MTBC in PTB and *Pseudomonas aeruginosa* in part of the Non-PTB group, are expected to reflect the underlying etiological diagnoses rather than a tuberculosis-specific microbiome signature. Therefore, the present findings should be interpreted as descriptive differences in lower-airway microbial community composition rather than as a validated diagnostic classifier. Larger studies incorporating clinically homogeneous disease controls will be required to determine whether reproducible non-MTBC microbial differences can be identified between PTB and clinically homogeneous comparator groups.

Several limitations should be acknowledged. The relatively small sample size and single-center retrospective design may limit the generalizability of our findings. Independent validation in larger, multicenter cohorts will be required to confirm whether the observed microbial features are reproducible across different populations. Potential confounding effects of mixed infections could not be fully excluded, which may weaken interpretations regarding microbial differences associated with PTB. Because smoking prevalence differed between groups, the Non-PTB group was clinically heterogeneous, and covariate-adjusted microbiome analyses were not performed, residual confounding cannot be excluded and the observed non-MTBC microbial differences cannot be attributed specifically to tuberculosis. Differential-abundance analyses were exploratory and did not use a compositionality-aware, FDR-controlled multivariable framework. Therefore, identified taxa should be interpreted as between-group differences in relative abundance rather than PTB biomarkers. Although repeated antibiotic exposure was excluded according to the predefined criterion, potential residual effects of less frequent prior antibiotic use on the pulmonary microbiome cannot be completely excluded. Moreover, the cross-sectional design precludes assessment of temporal dynamics between microbiome shifts and disease progression.

## 5 Conclusions

In summary, this BALF metagenomic study identified modest but statistically detectable differences in lower-airway microbial community composition between PTB and a heterogeneous group of non-PTB pulmonary diseases. Beyond the expected predominance of disease-associated pathogens, several accompanying microbial taxa differed between groups, providing a descriptive view of the differences in lower-airway microbial composition between these clinical groups. These findings should be considered hypothesis-generating and require validation in larger cohorts with clinically homogeneous comparator groups.

## Data Availability

The datasets presented in this study can be found in online repositories. The names of the repository/repositories and accession number(s) can be found below: https://db.cngb.org/cnsa/, CNP0007634.
