# Supplementary material for: Characterization of lower-airway microbial communities in pulmonary tuberculosis by BALF metagenomic sequencing
Source: Front Cell Infect Microbiol. 2026 Oct 23;16:1938581. doi: 10.3389/fcimb.2026.1938581 (PMC13739553; doi:10.3389/fcimb.2026.1938581)
Supplement: Supplementary file 1 [file DataSheet1.pdf]

Supplementary Material

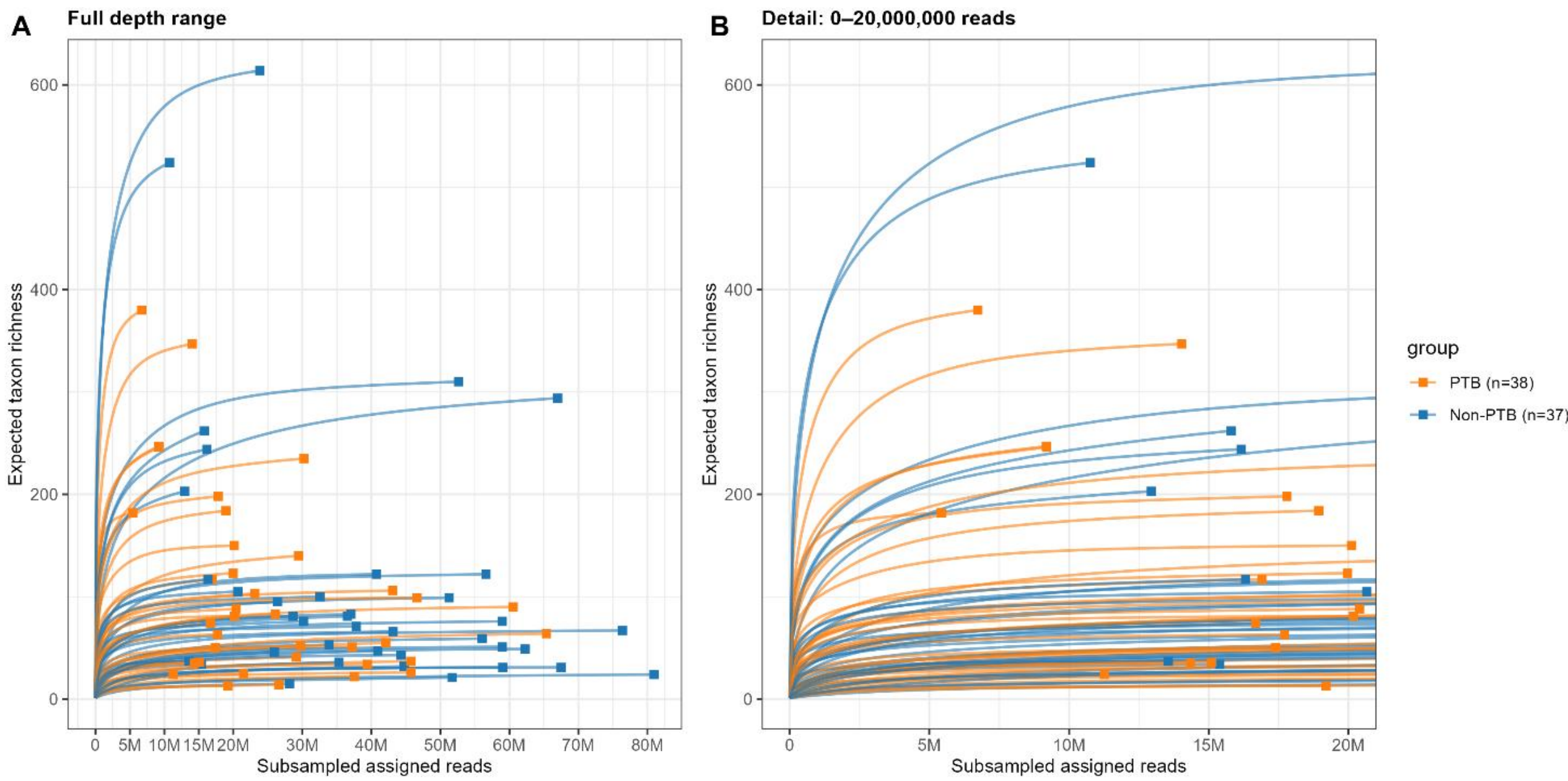

Figure S1. Read-based rarefaction curves of taxon richness. Expected taxon richness was analytically computed from raw taxon-assigned reads under sampling without replacement. (A) Full-depth rarefaction plot. (B) Expanded view for read depths ranging from 0 to 20 million reads with linear x-axis.

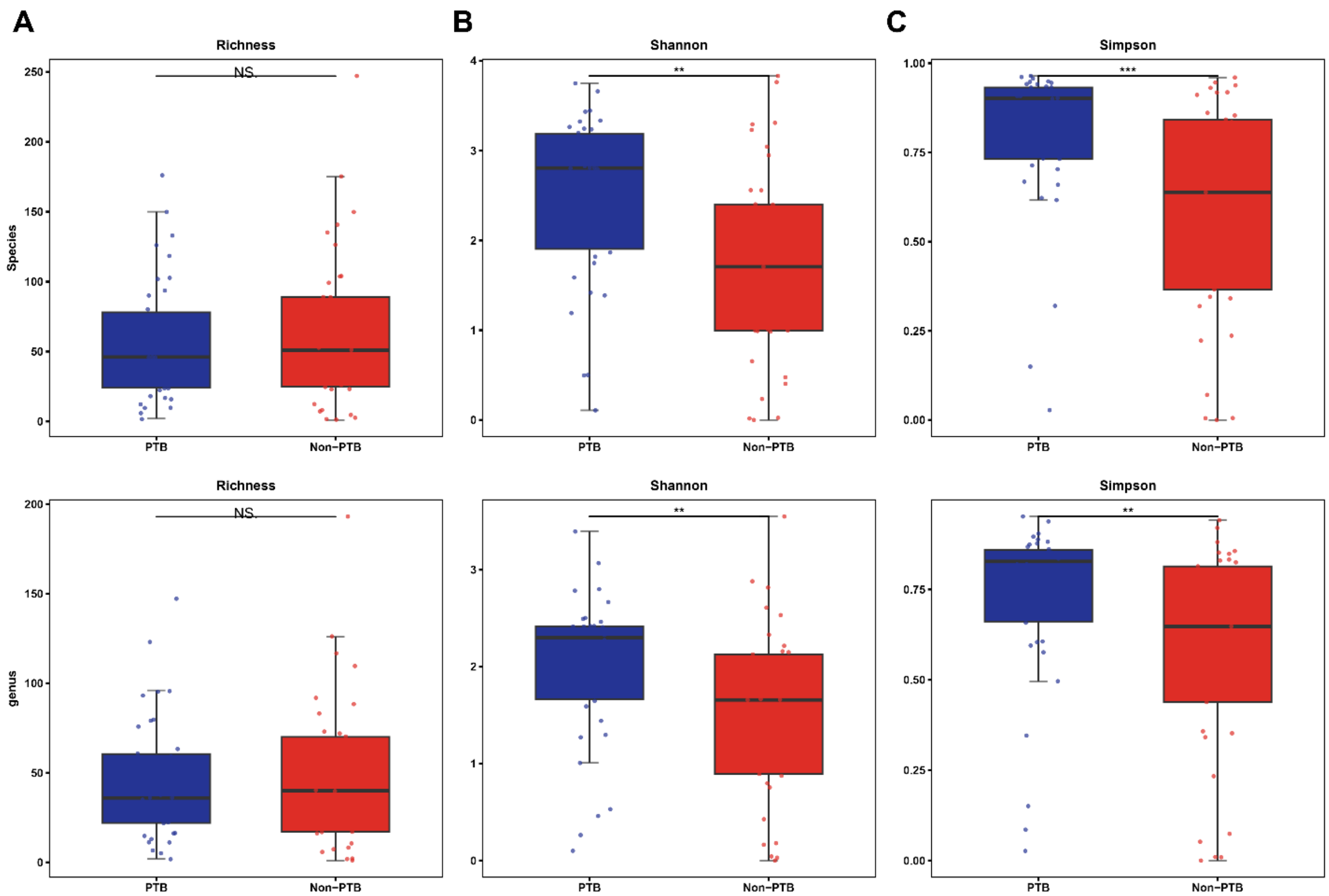

Figure S2. Alpha diversity at species and genus levels in PTB and Non-PTB groups after MTBC removal. (A) Richness index; (B) Shannon index; (C) Simpson index.

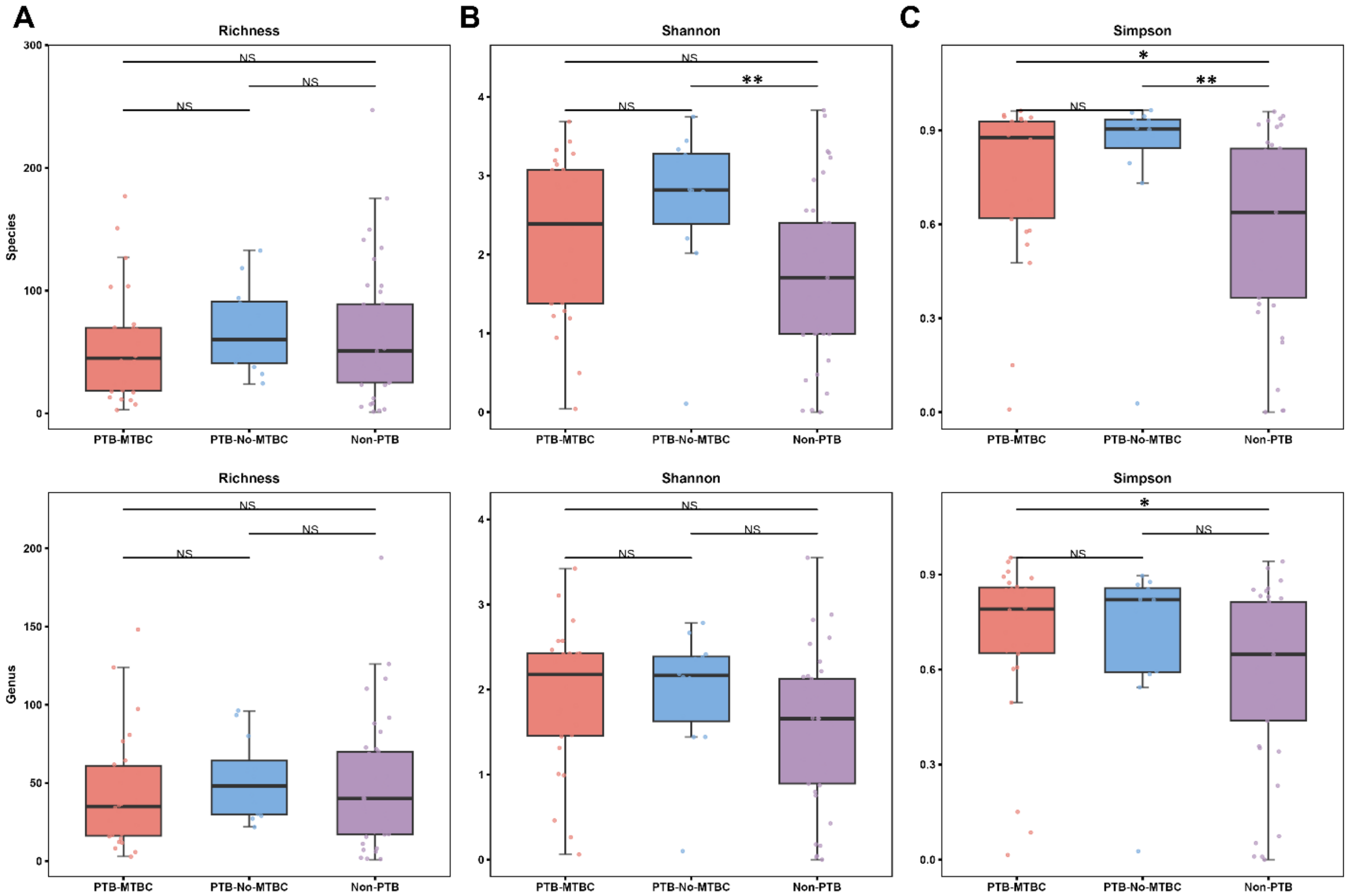

Figure S3. Comparison of alpha diversity at species and genus levels among PTB-MTBC, PTB-No-MTBC and Non-PTB groups. (A) Richness index; (B) Shannon index; (C) Simpson index.

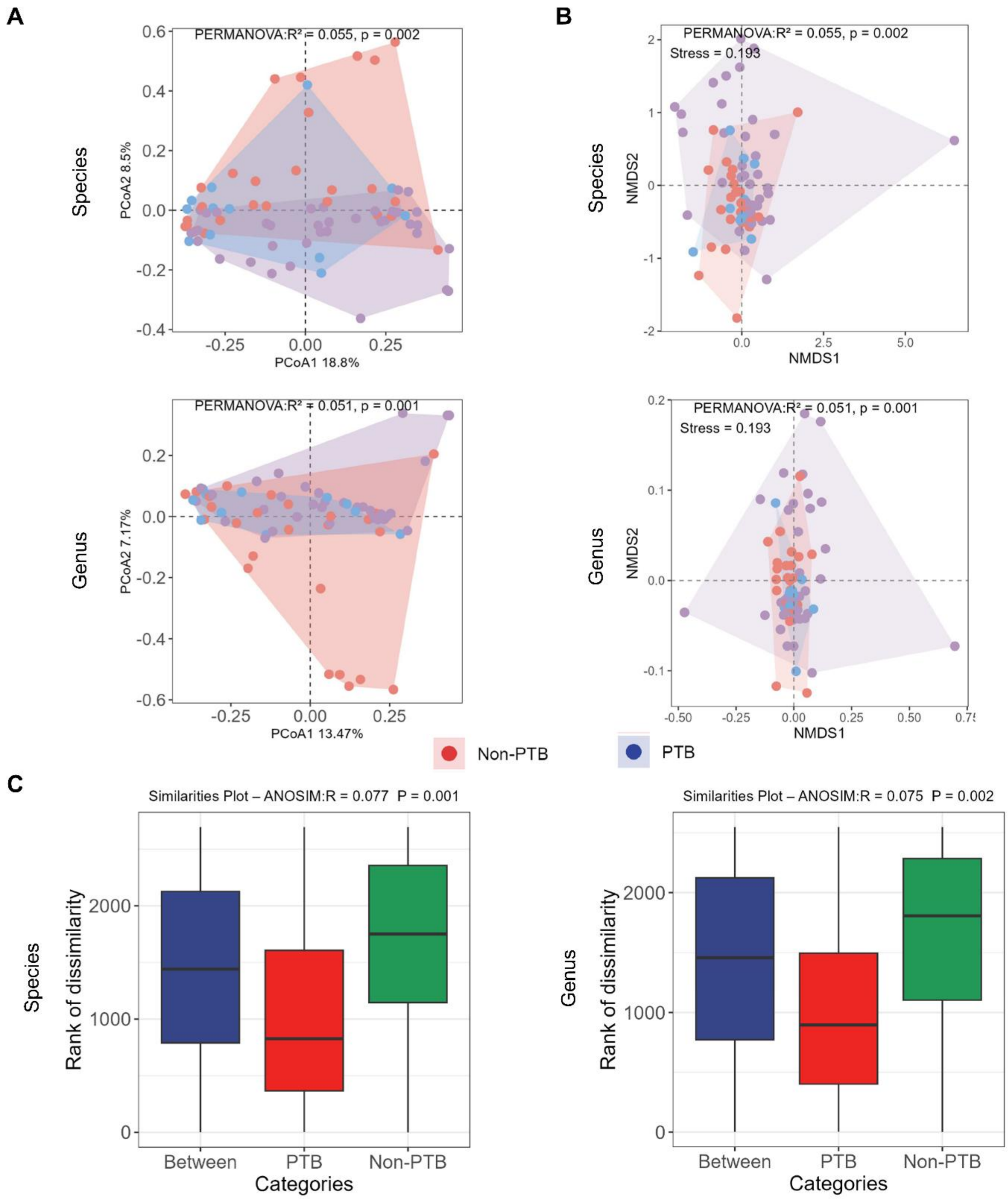

Figure S4. Comparison of beta diversity at species and genus levels between PTB and Non-PTB groups after MTBC removal. (A) PCoA analysis based on Bray-Curtis distance; (B) NMDS analysis based on Bray-Curtis distance; (C) ANOSIM analysis based on Bray-Curtis distance for intergroup comparison.

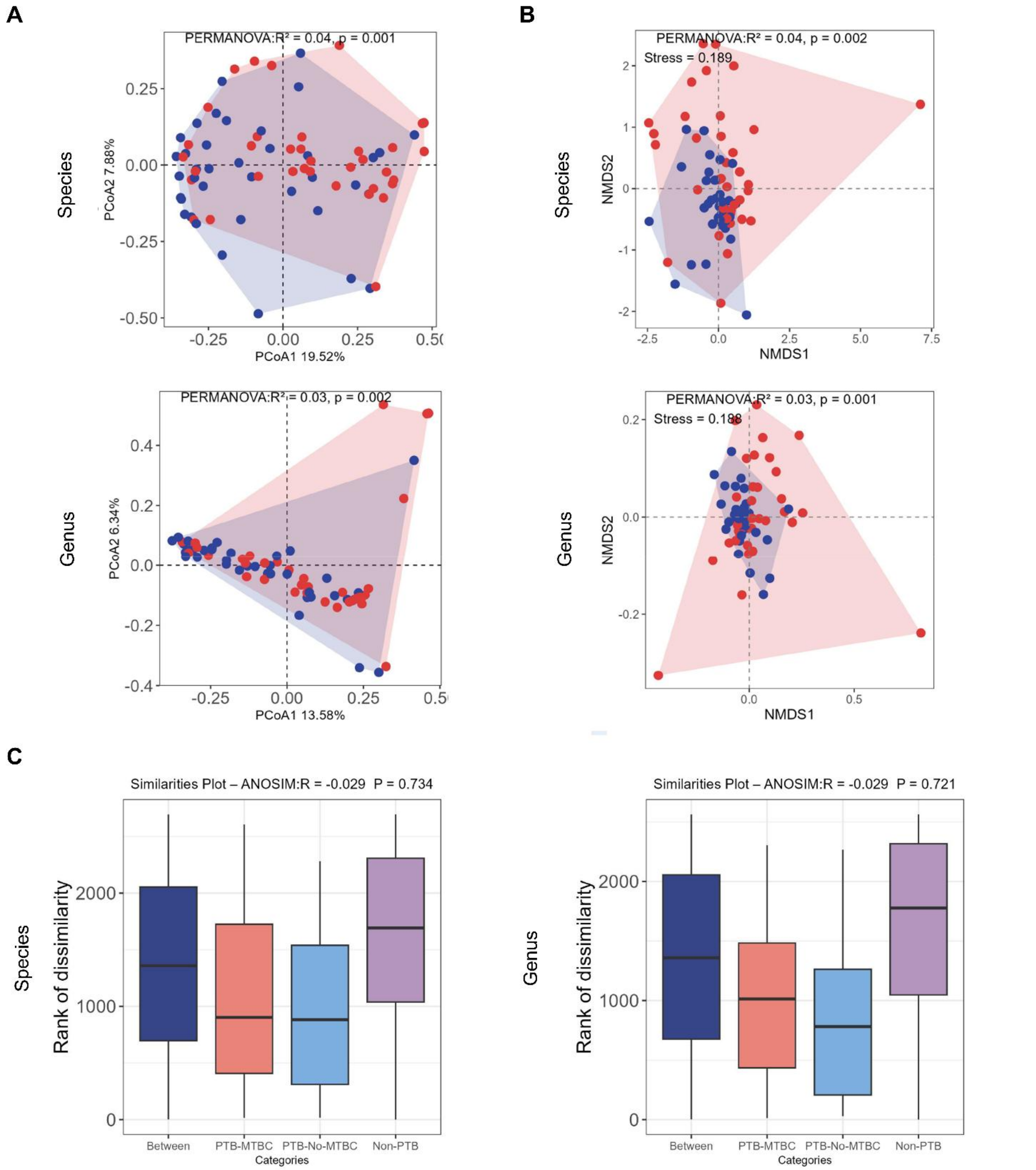

Figure S5. Comparison of beta diversity at species and genus levels among PTB-MTBC, PTB-No-MTBC and Non-PTB groups. (A) PCoA analysis based on Bray-Curtis distance; (B) NMDS analysis based on Bray-Curtis distance; (C) ANOSIM analysis based on Bray-Curtis distance for intergroup comparison.

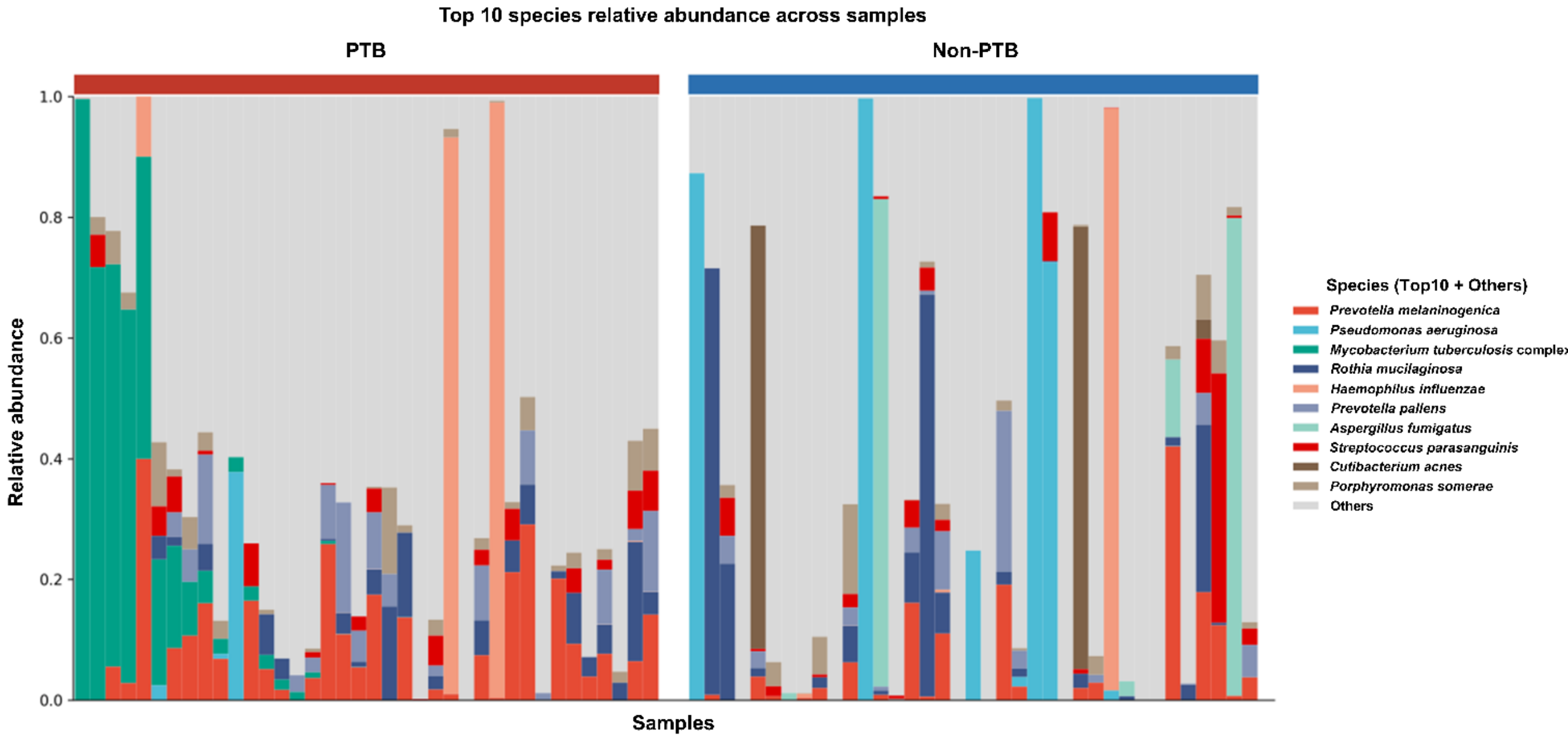

Figure S6. Sample-level relative abundance profiles of dominant microbial species in PTB and Non-PTB BALF samples. Each stacked bar represents an individual BALF sample, with colors indicating the relative abundance of the top 10 species; all remaining taxa are grouped as "Others." Samples are stratified by clinical group to visualize between-patient heterogeneity in species-level microbial composition. Taxonomic nomenclature was standardized throughout the figure.

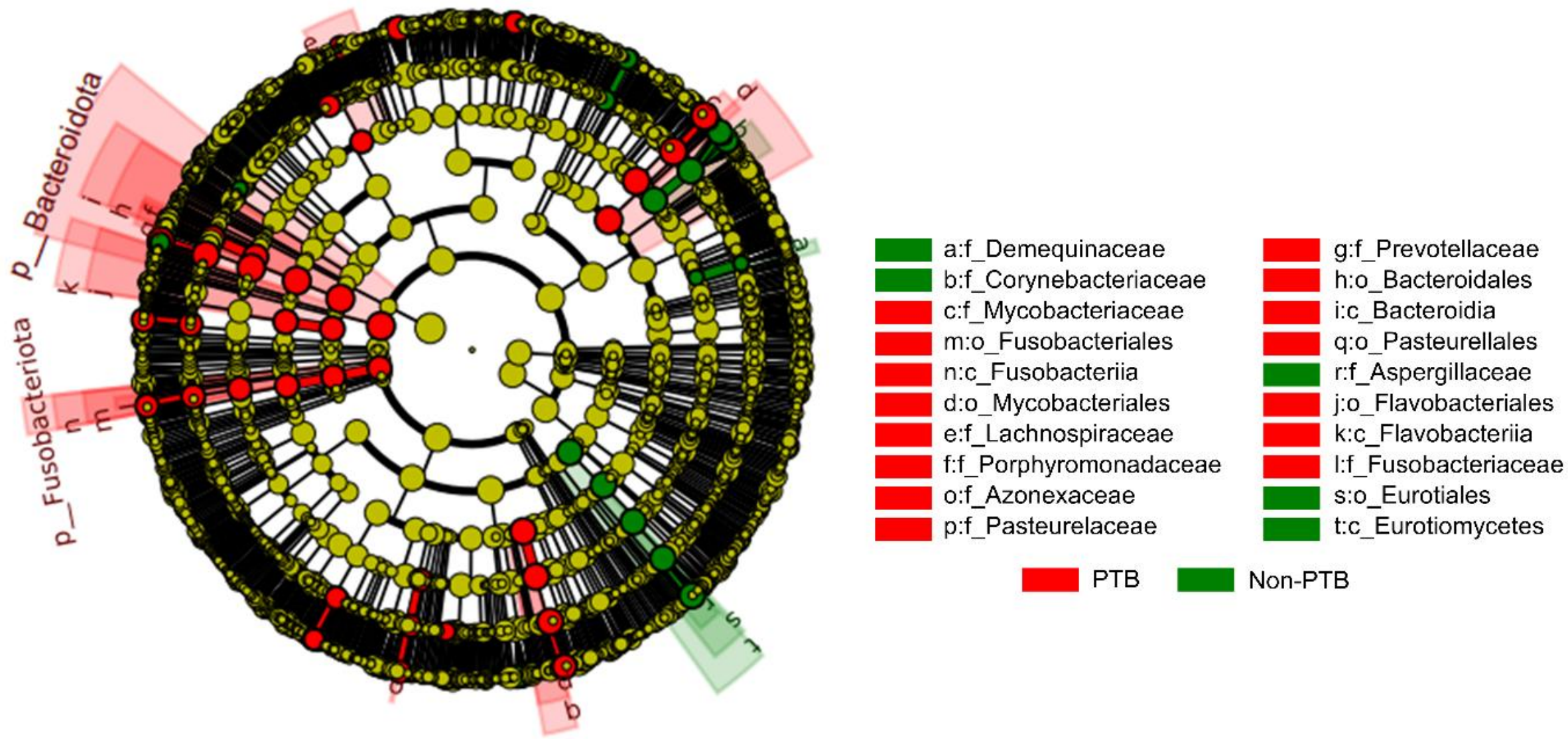

Figure S7. LEfSe cladogram of taxa showing differential relative abundance between PTB and Non-PTB groups. The cladogram shows the phylogenetic distribution of taxa identified by the exploratory LEfSe analysis. Circles represent taxonomic levels from phylum to species, and node size reflects relative abundance. Taxa identified by LEfSe are highlighted by group, whereas taxa not identified by LEfSe are shown in yellow. Node annotations correspond to the taxa listed in the figure legend.
